# Phosphorylation of PLIN3 by AMPK promotes dispersion of lipid droplets during starvation

**DOI:** 10.1007/s13238-018-0593-9

**Published:** 2018-11-14

**Authors:** Jianxi Zhu, Mingyang Xu, Yi Liu, Lisha Zhuang, Kejun Ying, Feng Liu, Dan Liu, Wenbin Ma, Zhou Songyang

**Affiliations:** 1 MOE Key Laboratory of Gene Function and Regulation, State Key Laboratory for Biocontrol, School of Life Sciences, Sun Yat-sen University, Guangzhou, 510275 China; 2 Guangzhou Regenerative Medicine and Health-Guangdong Laboratory (GRMH-GDL), Guangzhou, 510530 China; 3 Verna and Marrs McLean Department of Biochemistry and Molecular Biology, Baylor College of Medicine, One Baylor Plaza, Houston, TX 77030 USA

**Dear Editor,**

Lipid droplets (LDs) are dynamic lipid-storage organelles of storage depots and sources of essential substrates for myriad cellular processes and protect cells from lipotoxicity (Ohsaki et al., 2006). Disrupted LD and fat storage homeostasis has been linked to metabolic diseases such as atherosclerosis, obesity, and type II diabetes (Levin et al., 2001). Structurally, the core of neutral lipids in LDs is surrounded by a phospholipid monolayer and coated with specific proteins (Storey et al., 2011). Perilipin family of proteins are the predominant LD-associated proteins. In mammals, Perilipin 1 (PLIN1) is primarily expressed in adipose tissues and a major regulator of lipolysis in adipocytes (Kuo et al., 2017). PLIN2 and PLIN3 help coat LDs in most other cell types (Bulankina et al., 2009). Unlike PLIN1/2, PLIN3 targets primarily to nascent LDs and remains stable in the cytoplasm when not associated with LDs (Hocsak et al., 2010). It has emerged as a regulator of LD biogenesis and degradation (Bulankina et al., 2009).

The AMP-activated protein kinase (AMPK) is comprised of α, β and γ subunits and regulates cellular energy homeostasis (Carling et al., 1994). Activation of AMPK upon stress conditions such as glucose deprivation, occurs through AMP-γ subunit binding or Thr172 phosphorylation. Activated AMPK acts on targets in diverse pathways, from carbohydrate, protein and lipid metabolism to mitochondrial biogenesis, autophagy and cell growth (Fraser et al., 2013). Despite being a major cellular regulator of lipid metabolism (Dyck et al., 1999), direct targets of AMPK in LD homeostasis remain elusive. We report here that PLIN3 is a novel physiological AMPK substrate where phosphorylation by AMPK may help expose PLIN3 C-terminus to promote LD dispersion.

AMPK activation can promote LD dispersion during starvation or following addition of AMPK activators (Herms et al., 2015). To determine whether AMPK-regulated activation of perilipin family proteins might be key to LD dispersion, we performed Bi-molecular Fluorescence Complementation (BiFC) assays to detect AMPK-perilipin interaction using YFPn-tagged AMPKα1 and YFPc-tagged perilipins (Figs. 1A and S1). Interestingly, AMPKα1 could interact with PLIN3, but not PLIN2/4/5 (Fig. 1B). This interaction was further confirmed by co-Immunoprecipitation, where PLIN3 not only co-immunoprecipitated with AMPKα1, but also AMPKβ2 and AMPKγ1 (Fig. 1C). Furthermore, PLIN3 appeared to also associate with LKB1, a key activator of AMPK.

**Figure 1:**
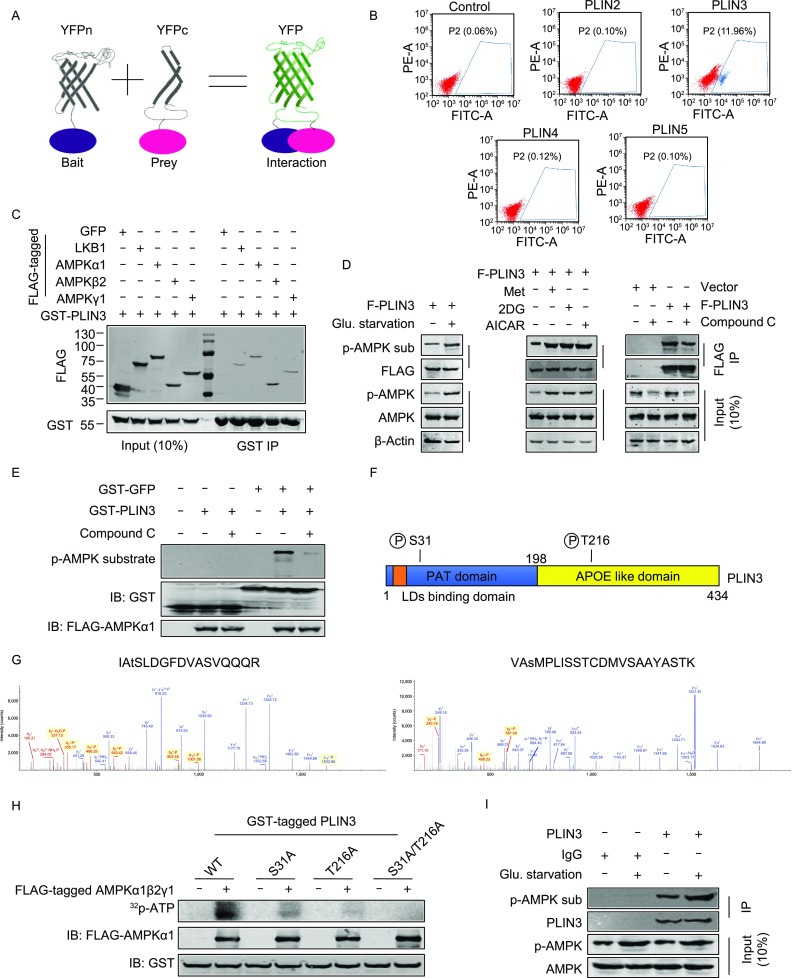
**PLIN3 interact with AMPK complex**. (A) In BiFC assay, two proteins (bait and prey) are tagged respectively with the N- and C-terminal halves of Venus YFP (YFPn and YFPc). Interaction between the two proteins will bring the YFP fragments together, resulting in co-folding and fluorescence complementation that can be detected by flow cytometry and microscopy in live cells. (B) YFPn targeted AMPKα1 and YFPc targeted PLINs are stably expressed in HTC75 cell. Only expressed YFPn targeted AMPKα1 as a negative control. The fluorescence detected by flow cytometer. (C) 293T cells co-express GST-tagged PLIN3 with LKB1, AMPKα1, AMPKβ2 and AMPKγ1, immunoprecipitation with anti-GST antibodies and western blotted using the Flag antibodies. (D) 293T cells expressing FLAG-tagged PLIN3 (F-PLIN3) were glucose (Glu.) starved (left), treated with AMPK activators Met, 2DG, or AICAR (middle), or incubated with the AMPK inhibitor compound C (right) for 16 h before being harvested for immunoprecipitation with anti-FLAG antibodies and western blotted using the indicated antibodies. Antibodies against β-actin served as loading control. (E) For *in vitro* kinase assays, bacterially purified GST-tagged PLIN3 was incubated with the AMPK complex immunoprecipitated from 293T cells that co-expressed AMPK α1, β2 and γ1 and had been treated with or without the AMPK inhibitor compound C for 16 h. The reaction mixtures were resolved by SDS-PAGE and probed with the indicated antibodies. (F) The function domain and phosphorylation sites of AMPK in PLIN3. (G) IP-Mass Spec detects the phosphorylation peptide sequence and indicates *in vivo* phosphorylation of S31 and T216 on PLIN3. (H) Bacterially purified GST-tagged wildtype (WT) and phosphorylation mutants (S31A, T216A, or S31A/T216A) of PLIN3 were incubated with the AMPK complex immunoprecipitated from 293T cells co-expressing AMPK α1, β2 and γ1 for *in vitro* kinase assays in the presence of ^32^P-ATP. Samples were resolved by SDS-PAGE and probed with the indicated antibodies. PLIN3 phosphorylation was detected by autoradiography

We then tested whether glucose depletion could induce AMPK-dependent phosphorylation of PLIN3 using cells ectopically expressing FLAG-tagged PLIN3 and a phospho-AMPK substrate motif antibody. Upon glucose depletion, a two-fold increase in PLIN3 phosphorylation was detected (Fig. 1D, left), similar to the degree of increase in AMPK phosphorylation. Several compounds are known AMPK activity modulators: the diabetes drug metformin (Met), the glycolysis inhibitor 2-deoxy-D-glucose (2DG), the AMPK activator AICAR, and the potent and highly selective inhibitor dorsomorphin (compound C). Consistent with PLIN3 being an AMPK substrate, when cells transiently expressing FLAG-PLIN3 were treated with AMPK activators, increased PLIN3 phosphorylation was evident (Fig. 1D, middle). In contrast, PLIN3 phosphorylation decreased in compound C treated cells (Fig. 1D, right). Next, we carried out *in vitro* kinase assays using bacterially purified GST-PLIN3 and AMPK complex proteins immunoprecipitated from 293T cells co-expressing AMPK α1, β2 and γ1 (Fig. 1E). Here, recombinant PLIN3 could be strongly phosphorylated by the purified AMPK complex and compound C treatment greatly diminished this phosphorylation. These results indicate that PLIN3 is a potential direct substrate of AMPK.

Residues S31 and T216 of PLIN3 are possible AMPK *in vivo* phosphorylation sites based on GPS3.0 (http://gps.biocuckoo.org/) (Fig. 1F). Mass spectrometry sequencing of *in vitro* phosphorylated GST-PLIN3 confirmed that both residues were indeed phosphorylated (Figs. 1G and S2). We then performed *in vitro* kinase assays in the presence of γ-^32^p- ATP, using wildtype and Ala mutants (S31A and T216A) of PLIN3. Again, PLIN3 phosphorylation was readily detectable (Fig. 1H), and Ala mutation of either residue led to decreased PLIN3 phosphorylation. In fact, mutation of both residues completely abolished PLIN3 phosphorylation, indicating that residues S31 and T216 are phosphorylated by AMPK. To examine endogenous PLIN3, we again used the phospho-AMPK substrate antibody, which could detect S31/T216 phosphorylation in recombinant PLIN3 (Fig. S3). Consistent with our data on ectopically expressed PLIN3, phosphorylation of endogenous PLIN3 (brought down with anti-PLIN3 antibodies) could be detected and the signal increased upon glucose starvation (Fig. 1I), suggesting that S31 and/or T216 are phosphorylated in response to AMPK activation *in vivo*.

To understand how AMPK-mediated PLIN3 phosphorylation regulates LD biology, we visualized LDs in glucose-starved cells with the BODIPY 493/503 dye. LDs can be categorized into states of smear, clumped, or dispersed (Herms et al., 2015) (Fig. S4). LD dispersion response varied significantly among different cell lines, with HTC75 and HEK293 showing more obvious dispersion than HeLa (Fig. 2A and 2B). HTC75 cells also exhibited time-dependent increase in LD dispersion upon starvation (Fig. S5A and S5B) and were therefore used for further studies. LD dispersion was obvious in HTC75 cells treated with AMPK activators AICAR or 2DG (Fig. S6A and S6B), indicating that AMPK activation is important for LD dispersion.

**Figure 2:**
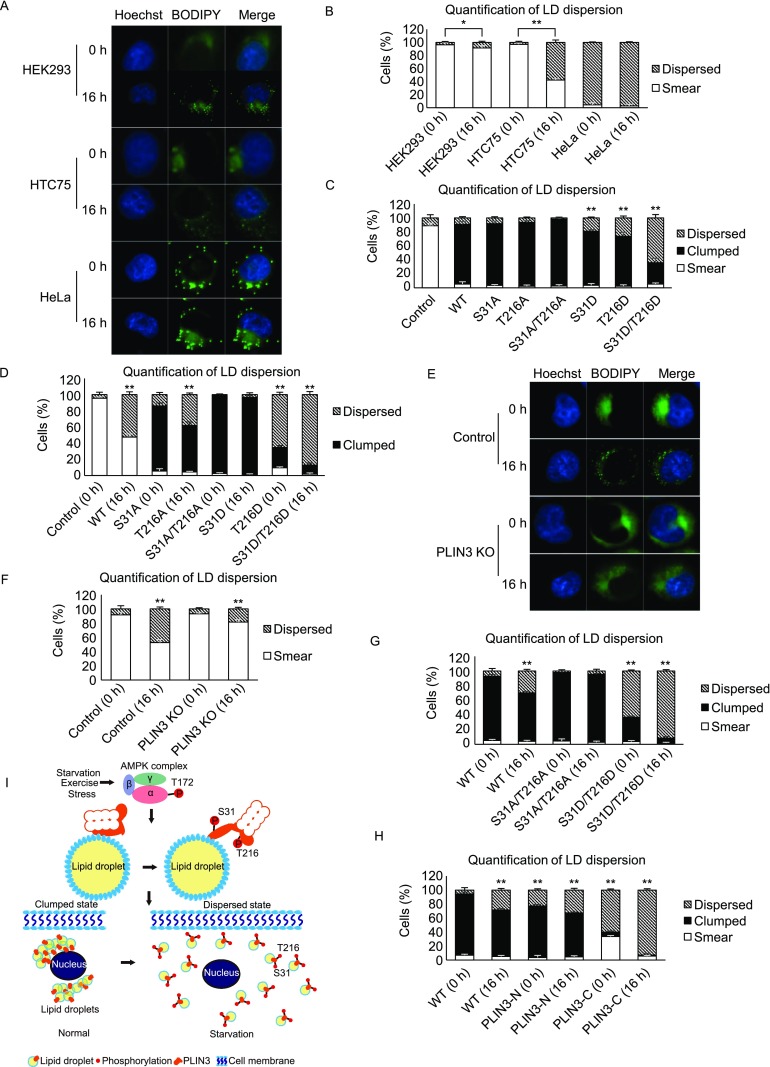
**AMPK phosphorylation of PLIN3 contributes to lipid droplet dispersion**. (A) HEK293, HTC75 and Hela cells were glucose starved for 16 h before being stained with the BODIPY 493/503 dye to label lipid droplets (green) and Hoechst 33342 to label DNA (blue). Cells with <10 dots were scored as having LD smears. Cells with ≥10 dots and each dot ≥20 μm from the nucleus were scored as having dispersed LD. Cells with ≥10 dots and each dot <20 μm from the nucleus or the volume of ≥1 dot being ≥10 times the dispersed size were scored as having clumped LDs. Should also include scale bars for all IF Figures. (B) Data from (A) were quantified and the percentages of cells with different LDs are plotted here. Error bars represent SD (*n* = 3). Statistical significance was calculated using the Student’s *t*-test. *P < 0.05. **P <0.01. (C) HTC75 cells ectopically expressing FLAG-tagged wildtype (WT), phosphorylation site mutants (S31A, T216A and S31A/T216A), or phosphomimic mutants (S31D, T216D and S31D/T216D) of PLIN3 were stained with BODIPY 493/503 (green) and Hoechst 33342 (blue). These cells quantified to derive the percentage of cells with different LDs. Error bars represent SD (*n* = 3). Statistical significance was calculated using the Student’s *t*-test. **P < 0.01. (D) HTC75 cells ectopically expressing FLAG-tagged wildtype (WT) PLIN3, PLIN3 S31A/T216A, or PLIN3 S31D/T216D were glucose starved for 16 h before being stained with BODIPY 493/503 (green) and Hoechst 33342 (blue). These cells quantified to derive the percentage of cells with different LDs. Error bars represent SD (*n* = 3). Statistical significance was calculated using the Student’s *t*-test. **P < 0.01. (E) Parental and PLIN3 KO HTC75 cells were glucose starved for 16 h and then stained with BODIPY 493/503 (green) and Hoechst 33342 (blue). (F) Data from (E) were quantified to derive the percentage of cells with dispersed LD. Error bars represent SD (*n* = 3). Statistical significance was calculated using the Student *t*-test. **P < 0.01. (G) PLIN3 KO cells expressing FLAG-tagged wildtype (WT) PLIN3, PLIN3 phosphorylation double mutant (S31A/T216A), or phospho-site mimic S31D/T216D were glucose starved for 16 h and then stained with BODIPY 493/503 (green) and Hoechst 33342 (blue). These cells were quantified to derive the percentage of cells with dispersed LD. Error bars represent SD (*n* = 3). Statistical significance was calculated using the Student’s *t*-test. *P < 0.05. **P < 0.01. (H) PLIN3 KO cells expressing FLAG-tagged wildtype (WT) PLIN3, PLIN3 N (residues 1–198) and PLIN3 C (residues 199–434) were glucose starved for 16 h and then stained with BODIPY 493/503 (green) and Hoechst 33342 (blue). These cells were quantified to derive the percentage of cells with dispersed LD. Error bars represent SD (*n* = 3). Statistical significance was calculated using the Student’s *t*-test. **P < 0.01. (I) Model of AMPK signaling through PLIN3 in lipid droplet response. Under normal conditions, the C-terminus of PLIN3 is blocked by its N-terminus, and lipid droplets are clumped near the nucleus. Upon starvation (and/or other signals), AMPK is activated (e.g., by low ATP/AMP ratios) and phosphorylates PLIN3 on residues S31 and T216. These phosphorylation events result in conformational changes of PLIN3 that unmask its C-terminal domain. Clumped lipid droplets then disperse from around the nucleus to the entire cytoplasm

We first generated HTC75 cells stably expressing wildtype PLIN3 and phosphorylation mutants of PLIN3 (S/T to A or D) (Fig. S7A). S/T to A mutations disrupt AMPK phosphorylation of PLIN3 and led to LD clustering rather than dispersion in >85% of the cells (Figs. 2C and S7B), whereas S/T to D mutations mimic AMPK phosphorylation and resulted in increased LD dispersion with ~62.5% of the cells expressing the double mutant (PLIN3 S31D/T216D) displaying LD dispersion. When we stained glucose-depleted cells expressing wildtype PLIN3 vs. double phosphorylation site mutants, LD dispersion could be observed in both control and WT PLIN3-expressing cells (Figs. 2D and S8). Notably, LD dispersion was completely abolished in S31A/T216A expressing cells, while increased LD dispersion was evident in S31D/T216D expressing cells. These observations suggest that PLIN3-mediated regulation of LD dynamics is modulated by AMPK phosphorylation at residues S31 and T216.

Next, we utilized the CRISPR-Cas9 technology to probe the function of endogenous PLIN3. Given the heterogeneity in LD response even within a single cell line, we generated pooled PLIN3 KO HTC75 cells rather than single KO cell clones (Fig. S9A, S9B and Supplementary Materials). Compared with parental cells, decreased LD dispersion was detectable in PLIN3 KO cells upon glucose starvation (from 47.2% to 18.4%) (Fig. 2E and 2F), which could be rescued by wildtype PLIN3 but not PLIN3 double phosphorylation mutant (S31A/T216A) (Figs. 2G, S10A and S10B). Furthermore, ectopic expression of PLIN3-S31D/T216D in KO cells resulted in enhanced LD dispersion (Figs. 2G, S10A and S10B). These data add additional support to the notion that AMPK phosphorylation of PLIN3 at S31 and T216 is critical for cellular lipid homeostasis.

S31 resides in the conserved N-terminal PAT domain that may immediate LD binding, and T216 resides in the C-terminal Apolipoprotein E (APOE)-like domain (with a four amphipathic α-helix bundle) involved in fatty acid transportation (Fig. 1F) (Hickenbottom et al., 2004). Both full-length PLIN3 and its C-terminus can reorganize lipids *in vitro*, which requires conformational changes in full-length PLIN3 to expose the C-terminal amphipathic helix bundle (Bulankina et al., 2009). Similar structural rearrangement has been documented in APOE-lipid interactions (Hickenbottom et al., 2004), raising the possibility that PLIN3 C-terminus may normally be folded inside PLIN3. Perhaps phosphorylation of S31 and T216 facilitates unfolding of the C-terminus, allowing it to participate in LD dispersion and fatty acid release. If so, expression of PLIN3 C-terminus alone should enhance LD dispersion. Under normal culture conditions, PLIN3 KO cells expressing PLIN3–N (residues 1–198) and full-length PLIN3 exhibited similar levels (>60%) of clustered LDs, whereas >60% of cells expressing PLIN3-C (residues 199–434) showed dispersed LDs (Fig. 2H, S11A and S11B); upon starvation, LD dispersion was blocked in PLIN3 KO cells ectopically expressing PLIN3-N, but enhanced in KO cells expressing PLIN3-C, supporting our hypothesis that PLIN3 phosphorylation by AMPK may help expose the PLIN3 C-terminus and promote LD dispersion and that the N- and C-terminus of PLIN3 may have opposing activities.

Functional differences between perilipin proteins may contribute to the distinct regulation of LDs in adipocytes (PLIN1-coated) vs. non-adipocyte cells (PLIN2/3-coated) (Patel et al., 2014). Perilipins are modulated by post-translational modifications, such as PKA phosphorylation of PLIN1/5 (Sanders et al., 2015), AMPK-dependent phosphorylation of PLIN2 (Kaushik and Cuervo, 2016) and ubiquitination of PLIN2/3 (Kaushik and Cuervo, 2016). Here, we provide evidence that PLIN3 is a novel and direct substrate of AMPK and PLIN3 phosphorylation by AMPK promotes LD dispersion, which implicates PLIN3 in lipolysis pathways. PLIN3 and PLIN2 are less efficient than PLIN1 at inhibiting basal lipolysis (Patel et al., 2014; Sanders et al., 2015). In both human and mouse, PLIN3 increases after exercise, consistent with PLIN3 promoting lipolysis (Louche et al., 2013). In response to energy crises during exercise, AMPK activation is faster than adrenaline-promoted PKA activation, thus expediting signal transduction to meet the energy demand in muscle and other non-adipose tissues. Perhaps PLIN3 promotes lipogenesis and lipid storage under normal conditions, and facilitates lipolysis after AMPK phosphorylation.

Our study supports the model where AMPK is activated during stress conditions and phosphorylates substrates such as PLIN3. AMPK phosphorylation of PLIN3 induces conformational changes that release PLIN3 C-terminus to participate in dispersing LDs (Fig. 2I). LD dispersion facilitates lipolysis and beta-oxidation of fatty acids. Our findings have important implications for the development of new and effective therapies for metabolic diseases such as obesity, type II diabetes and atherosclerosis.

## Electronic supplementary material

Below is the link to the electronic supplementary material.

Supplementary material 1 (PDF 829 kb)
