# Supplementary material for: Phosphorylation of PLIN3 by AMPK promotes dispersion of lipid droplets during starvation
Source: Protein Cell. 2018 Nov 14;10(5):382–7. doi: 10.1007/s13238-018-0593-9 (PMC6468040; doi:10.1007/s13238-018-0593-9)
Supplement: Supplementary file 1 — Supplementary material 1 (PDF 829 kb) [file 13238_2018_593_MOESM1_ESM.pdf]

Supplementary Figure S1. Confirmation of expression level of YFPc targeted perilipin family proteins.

YFPc targeted different perilipin family proteins are stable expressed in HTC75 cell. Cells were resolved by SDS-PAGE and probed with the GFP antibodies.

Supplementary Figure S2. MS peptide of S31 and T216 in PLIN3.

Representative peptide sequencing data by MS/MS^{+}. Production mass spectra for the mass-selected protonated phoshporylated peptide VASMPLISSTCDMVSAAYASTK and IATSLDGFDVASVQQQR of PLIN3 are included. S31 and T216 colored in red were identified as a phosphorylation site. b and y ions found in the mass spectra.

Supplementary Figure S3. The specificity of AMPK substrate antibody.

293T cells expressing FLAG-tagged GFP and FLAG-tagged PLIN3(WT, S31A, T216A and S31A/T216A) being harvested for immunoprecipitation with anti-FLAG antibodies and western blotted using the p-AMPK substrate antibodies.

Supplementary Figure S4. Different states of lipid droplets

Cells were stained with the BODIPY 493/503 dye to label lipid droplets (green) and Hoechst 33342 to label DNA (blue). Cells with <10 dots were scored as having LD smears. Cells with ≥10 dots and each dot ≥20μm from the nucleus were scored as having dispersed LD. Cells with ≥10 dots and each dot <20μm from the nucleus or the volume of ≥1 dot being ≥10 times the dispersed size were scored as having clumped LDs.

Supplementary Figure S5. Lipid droplets disperse after depletion of glucose and pyruvic acid in HTC75 cells.

A. HTC75 culture without glucose and pyruvic acid at 2h, 4h, 8h, 12h and 16h. Then stained with BODIPY (LD, green) and Hoechst (nucleus, blue).
B. Data from (A) were quantified to derive the percentage of cells with different LDs. Error bars represent SD (n=3).

Supplementary Figure S6. Treatment of AICAR and 2DG induce LDs dispersion.

A. HTC75 culture with AICAR and 2DG at 0h and 16h. Then stained with BODIPY (LD, green) and Hoechst (nucleus, blue).
B. Data from (A) were quantified to derive the percentage of cells with different LDs. Error bars represent SD (n=3).

Supplementary Figure S7. Phosphorylation of PLIN3 on S31 and T216 is important for LDs dispersion

A. HTC75 cells ectopically expressing FLAG-tagged wildtype (WT), phosphorylation site mutants (S31A, T216A, and S31A/T216A), or phosphomimic mutants (S31D, T216D, and S31D/T216D) of PLIN3 were harvested for western blotted using the GFP antibodies. Antibodies against β-actin served as loading control.

B. Cells from (A) were stained with BODIPY 493/503 (green) and Hoechst 33342 (blue).

Supplementary Figure S8. Overexpression of Wild type and mutant PLIN3 affect LDs states after cell starvation

HTC75 cells ectopically expressing FLAG-tagged wildtype (WT) PLIN3, PLIN3 S31A/T216A, or PLIN3 S31D/T216D were glucose starved for 16 hours before being stained with BODIPY 493/503 (green) and Hoechst 33342 (blue)

Supplementary Figure S9. Establishment and characterization of PLIN3 KO HTC75 cell line.

A. Two gRNA sequences targeting the exon 1 and exon 8 of PLIN3. The green square represent the exon of PLIN3. The phosphorylation sites S31 and T216 are respectively at PAT domain and APOE domain of PLIN3

B. Endogenous PLIN3 in WT and KO HTC75 cells were detected by PLIN3 antibody. β-Actin was used as reference.

Supplementary Figure S10. LD states after overexpressing Wild type and mutant PLIN3 in PLIN3 KO cells.

A. PLIN3 KO cells expressing FLAG-tagged wildtype (WT) PLIN3, PLIN3 phosphorylation double mutant (S31A/T216A), or phospho-site mimic S31D/T216D were harvested for western blotted using the GFP antibodies. Antibodies against -actin served as loading control.

B. Cells from (A) were glucose starved for 16 hours and then stained with BODIPY 493/503 (green) and Hoechst 33342 (blue).

Supplementary Figure S11. LD states after overexpressed N-PLIN3, C-PLIN3 and mutations in PLIN3 KO cells.

A. PLIN3 KO cells expressing FLAG-tagged wildtype (WT) PLIN3, PLIN3 N (residues 1-198), and PLIN3 C (residues 199-434) were harvested for western blotted using the GFP antibodies. Antibodies against -actin served as loading control.

B. Cells form (A) were glucose starved for 16 hours and then stained with BODIPY 493/503 (green) and Hoechst 33342 (blue).

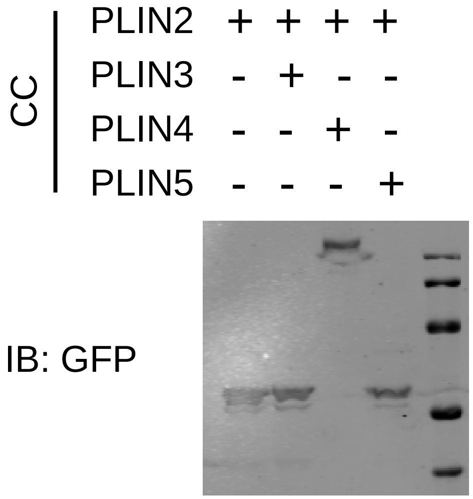
CC
PLIN2 + + + +
PLIN3 - + - -
PLIN4 - - + -
PLIN5 - - - +
IB: GFP

PLIN3 S31

VASMPLISSTCDMVSAAYASTK

#1	b^+	b^{2+}	Seq	y^+	y^{2+}	#2
1	100.07570	50.54149	V			22
2	171.11282	86.06005	A	2213.95292	1107.48010	21
3	338.11118	169.55923	S-Phospho	2142.91580	1071.96154	20
4	469.15168	235.07948	M	1975.91744	988.46236	19
5	566.20445	283.60586	P	1844.87694	922.94211	18
6	679.28852	340.14790	L	1747.82417	874.41572	17
7	792.37259	396.68992	I	1634.74010	817.87369	16
8	879.40462	440.20595	S	1521.65603	761.33165	15
9	966.43665	483.72196	S	1434.62400	674.29962	14
10	1067.48433	534.24580	T	1347.59197	623.77578	13
11	1170.49352	585.75040	C	1246.54429	572.27119	12
12	1285.52047	643.26387	D	1143.53510	514.75771	11
13	1416.56097	708.78412	M	1028.50815	449.23746	10
14	1515.62939	801.83435	V	897.46765	399.70325	9
15	1602.66142	837.35291	S	798.39923	356.18724	8
16	1673.69854	872.87147	A	711.36720	320.66868	7
17	1744.73566	954.40313	A	640.33008	285.15012	6
18	1907.79898	989.92169	Y	569.29296	203.61846	5
19	1978.83610	1033.43770	A	406.22964	168.09990	4
20	2065.86813	1083.96154	S	335.19252	124.58388	3
21	2166.91581		T	248.16049	74.06004	2
22			K	147.11281		1

PLIN3 T216

IATSLDGFDVASVQQQR

#1	b^+	b^{2+}	Seq	y^+	y^{2+}	#2
1	114.09135	57.54921	I			17
2	185.12847	93.06787	A	1801.81171	901.40949	16
3	365.14248	183.57488	T-Phospho	1730.77459	865.89093	15
4	453.17451	227.89089	S	1549.76058	775.38393	14
5	566.25858	283.63293	L	1462.72855	731.86791	13
6	681.28553	341.14640	D	1349.64448	675.32588	12
7	738.30700	369.65714	G	1234.61753	617.81240	11
8	885.37542	433.19135	F	1177.59606	589.30167	10
9	1000.40237	500.70482	D	1030.52764	515.76746	9
10	1099.47079	550.23903	V	915.50069	458.25398	8
11	1170.50791	585.75759	A	816.43227	408.71977	7
12	1257.53994	629.27361	S	745.39515	373.30131	6
13	1356.60836	678.80782	V	638.36312	329.68520	5
14	1484.66694	742.83711	Q	559.29470	280.15099	4
15	1612.72552	806.86640	Q	431.23612	216.21170	3
16	1740.78410	870.89569	Q	303.17754	152.09241	2
17			R	175.11896	86.06312	1

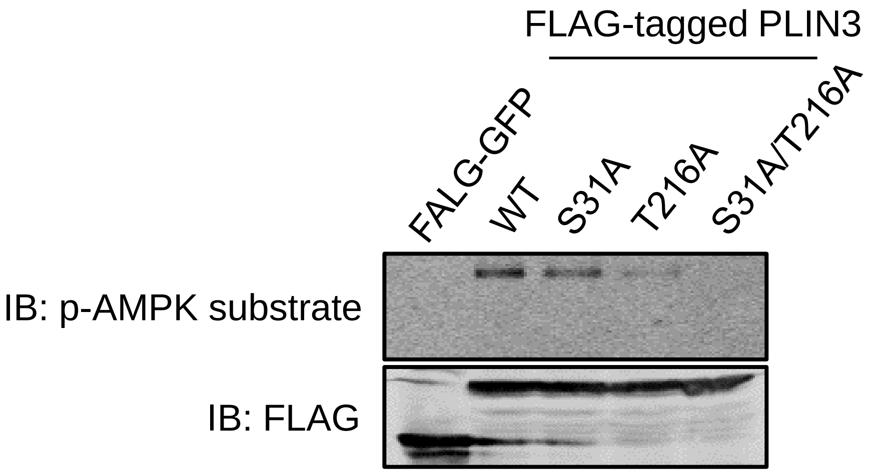
FLAG-tagged PLIN3
FALG-GFP
WT
S31A
T216A
S31A/T216A
IB: p-AMPK substrate
IB: FLAG

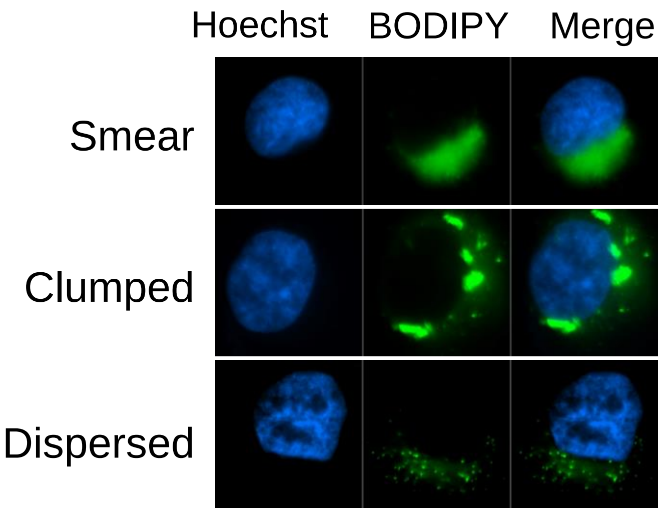
Hoechst
BODIPY
Merge
Smear
Clumped
Dispersed

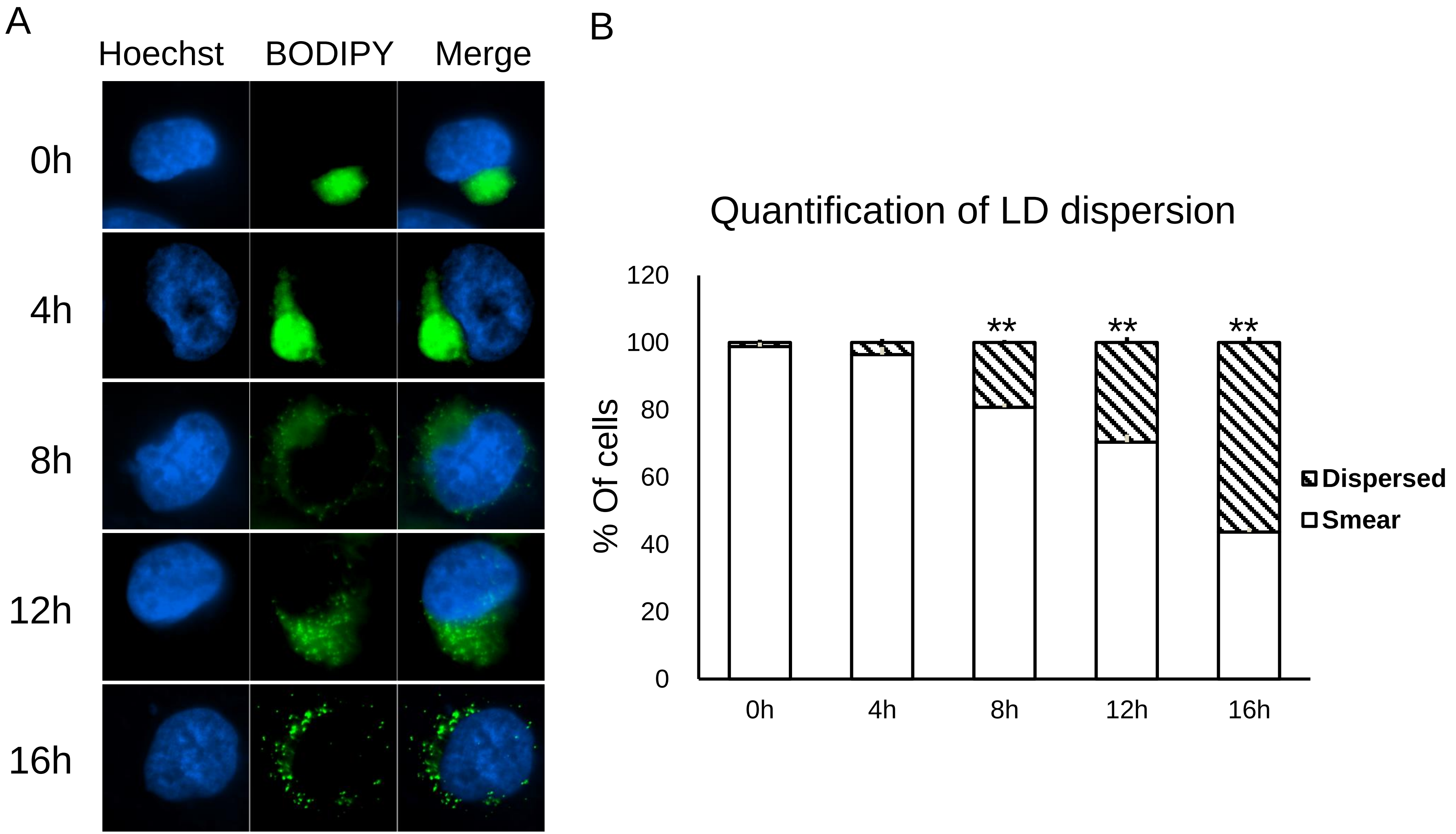
A
Hoechst
BODIPY
Merge
0h
4h
8h
12h
16h
B
Quantification of LD dispersion
% Of cells
120
100
80
60
40
20
0
**
**
**
0h
4h
8h
12h
16h
Dispersed
Smear

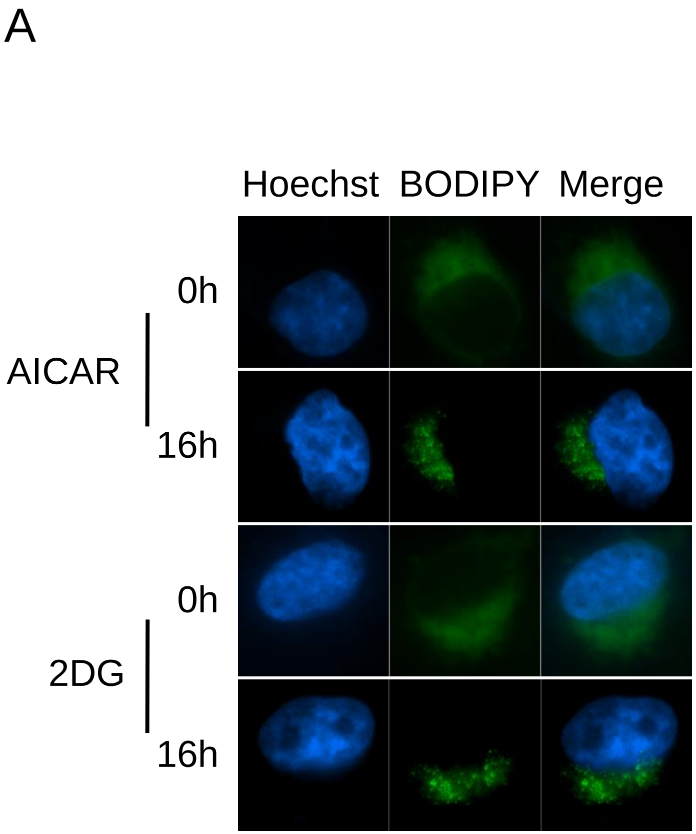
A
Hoechst
BODIPY
Merge
0h
AICAR
16h
0h
2DG
16h

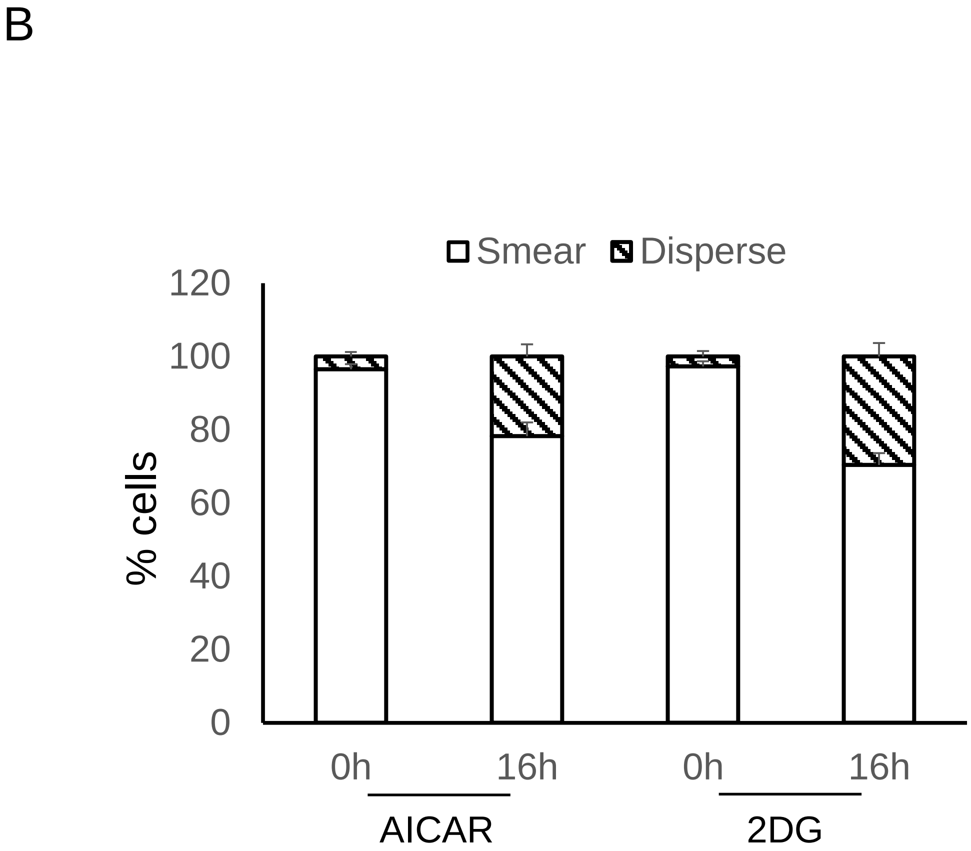
B
Smear
Disperse
120
100
80
60
40
20
0
% cells
0h
16h
0h
16h
AICAR
2DG

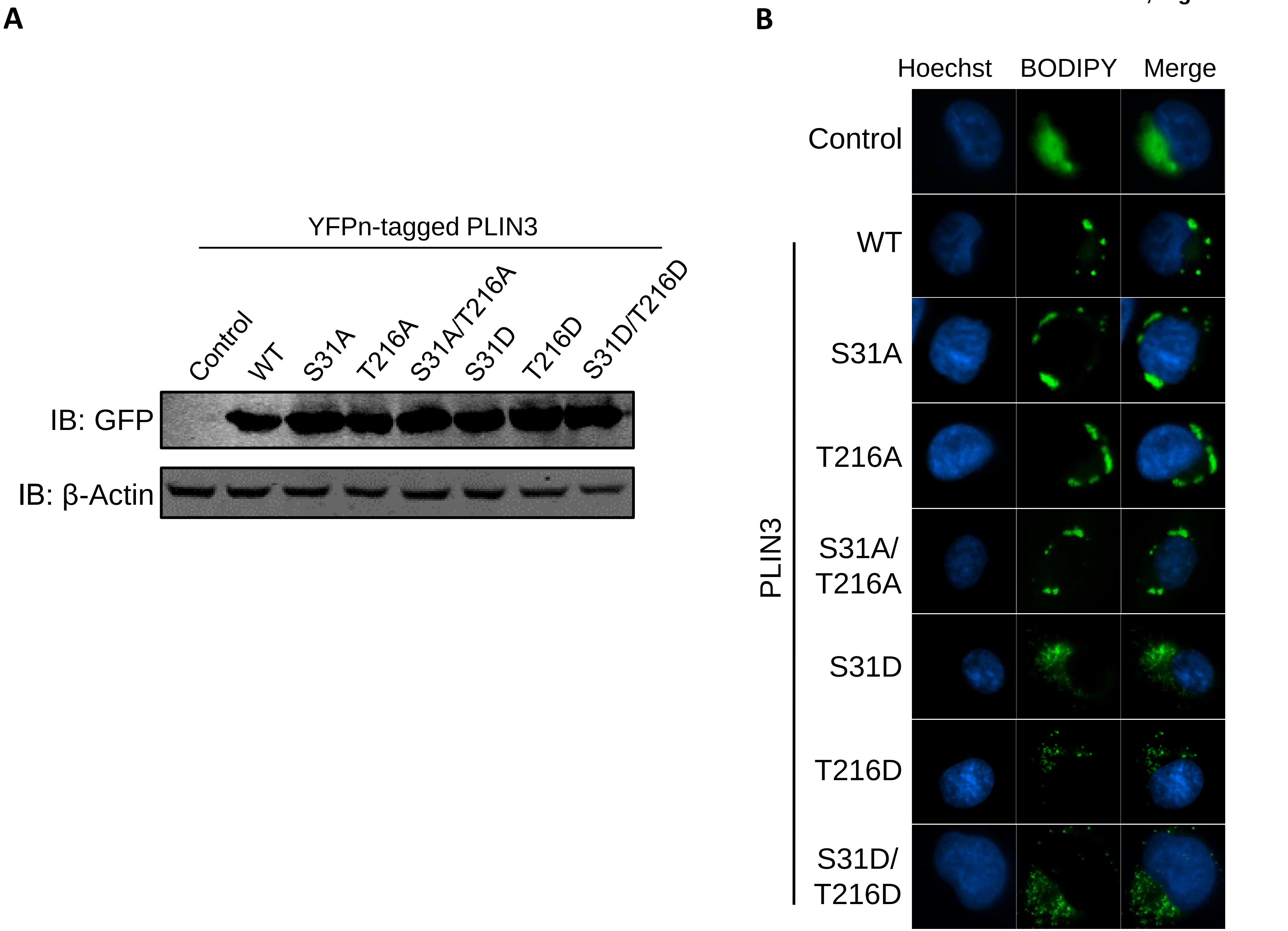
A
YFPn-tagged PLIN3
Control
WT
S31A
T216A
S31A/T216A
S31D
T216D
S31D/T216D
IB: GFP
IB: β-Actin
B
Hoechst
BODIPY
Merge
Control
WT
S31A
T216A
S31A/ T216A
S31D
T216D
S31D/ T216D
PLIN3

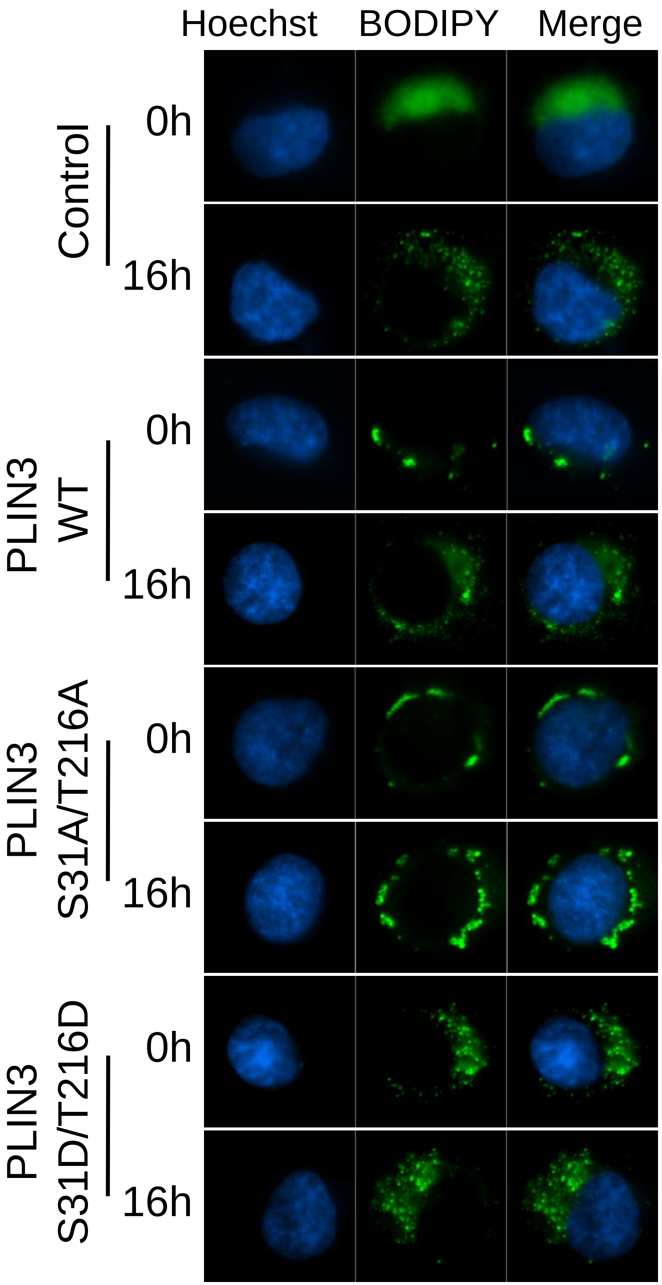
Hoechst
BODIPY
Merge
Control
0h
16h
PLIN3 WT
0h
16h
PLIN3 S31A/T216A
0h
16h
PLIN3 S31D/T216D
0h
16h

A

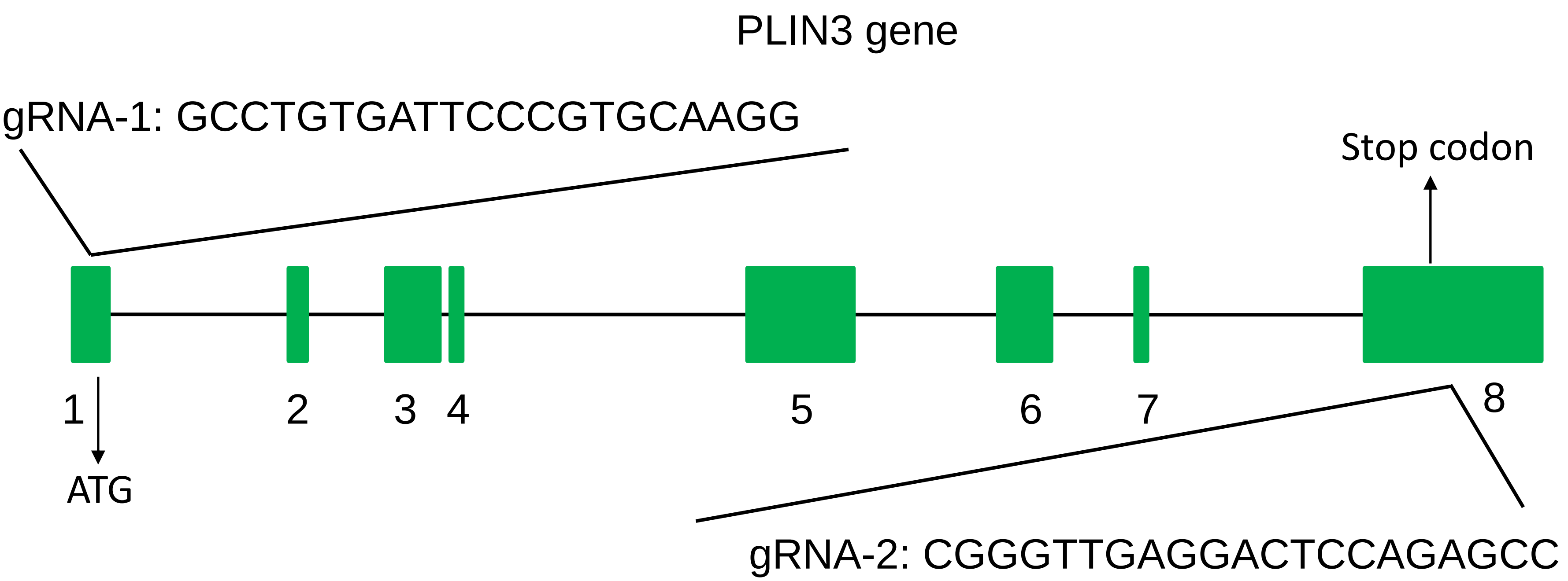
PLIN3 gene
gRNA-1: GCCTGTGATTCCCGTGCAAGG
Stop codon
1
2
3
4
5
6
7
8
ATG
gRNA-2: CGGGTTGAGGACTCCAGAGCC

B

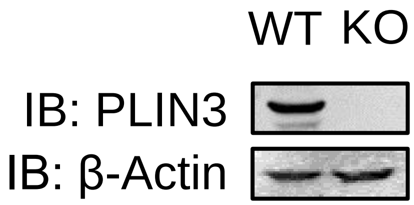
WT
KO
IB: PLIN3
IB: β-Actin

Zhu et al., Fig. S10A-B

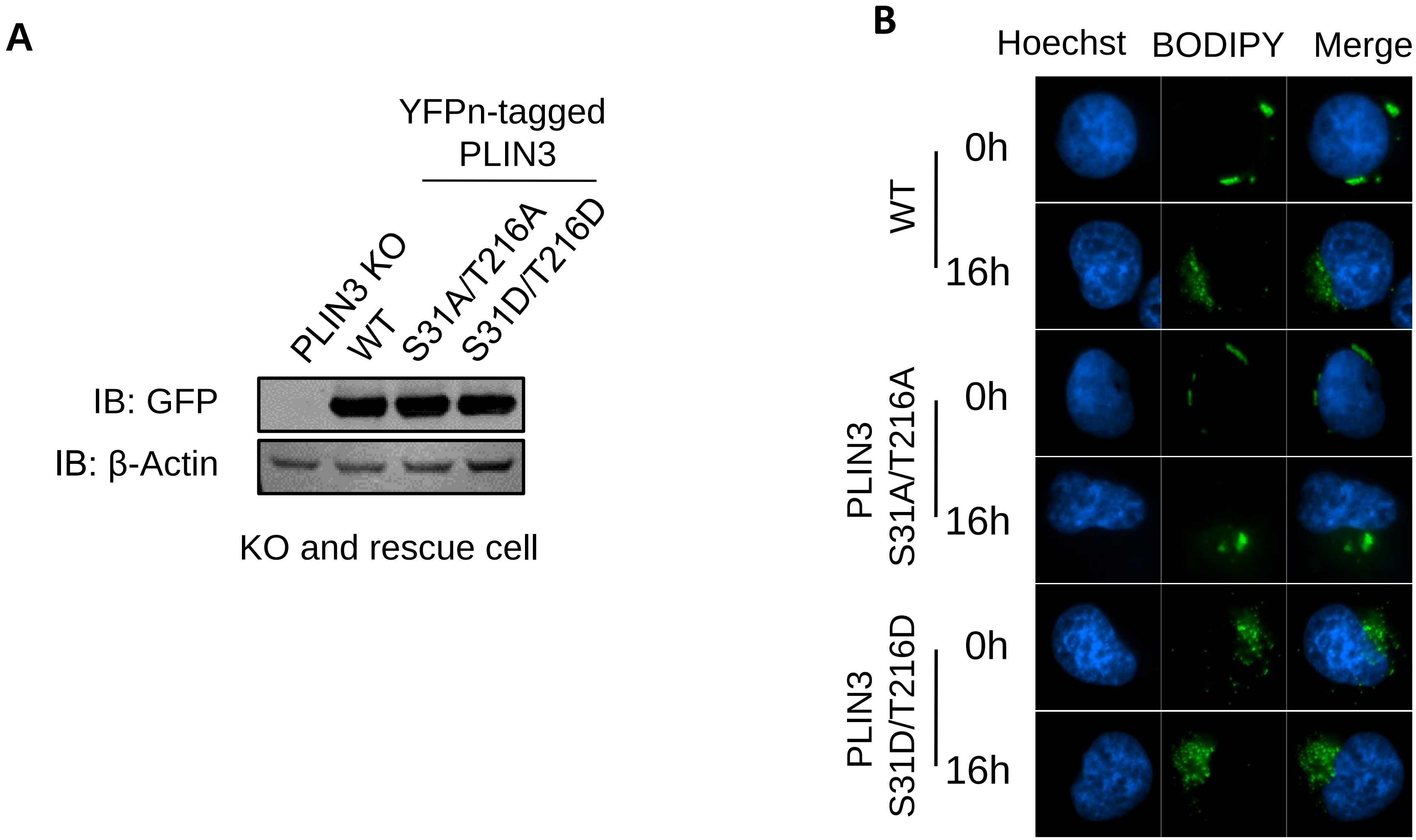

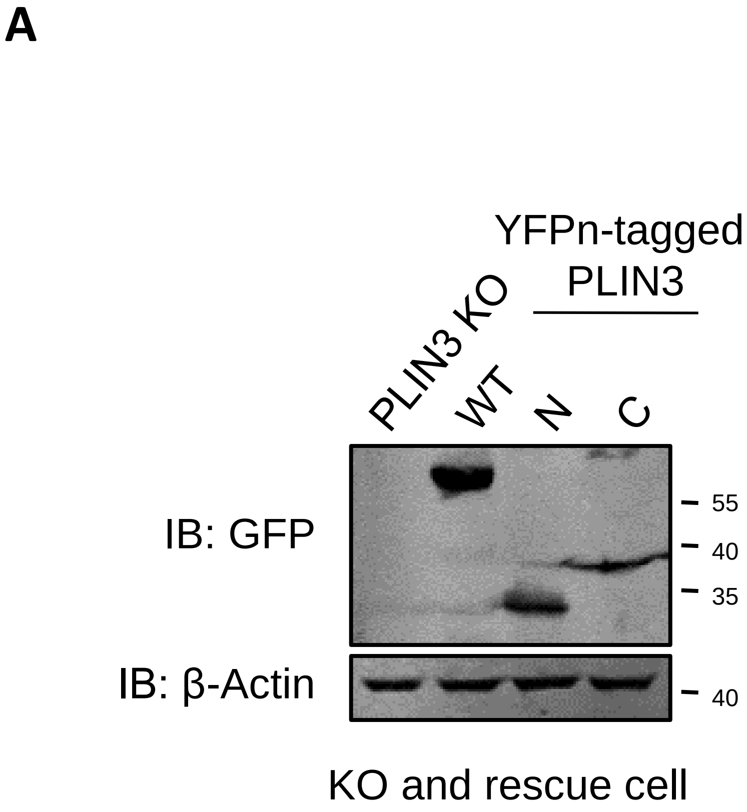
A
YFPn-tagged
PLIN3
PLIN3 KO
WT
N
C
IB: GFP
55
40
35
IB: β-Actin
40
KO and rescue cell

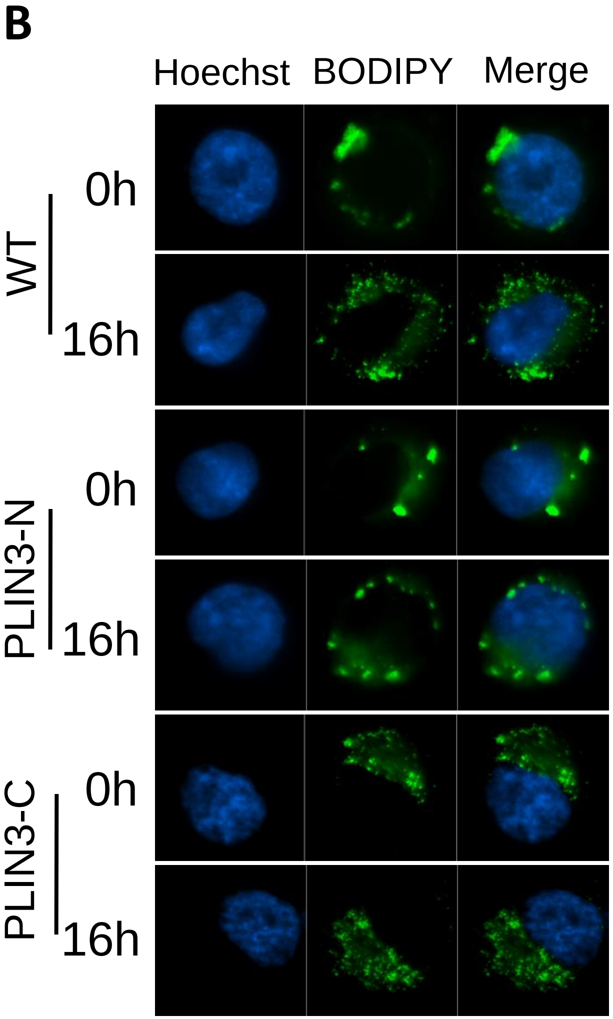
B
Hoechst
BODIPY
Merge
WT
0h
16h
PLIN3-N
0h
16h
PLIN3-C
0h
16h
